# Targeting EGF-receptor-signalling in squamous cell carcinomas of the head and neck

**DOI:** 10.1038/sj.bjc.6603566

**Published:** 2007-01-16

**Authors:** C W M Reuter, M A Morgan, A Eckardt

**Affiliations:** 1 Department of Hematology, Hemostaseology and Oncology, Hannover Medical School, Carl-Neuberg-Strasse 1, 30625 Hannover, Germany; 2 Department of Oral and Maxillofacial Surgery, Hannover Medical School, Carl-Neuberg-Strasse 1, 30625 Hannover, Germany

**Keywords:** EGF-receptor signalling, monoclonal antibody, tyrosine kinase inhibitor, SCCHN

## Abstract

Despite significant advances in the use of surgery, chemotherapy and radiotherapy to treat squamous cell carcinoma of the head and neck (SCCHN), prognosis has improved little over the past 30 years. There is a clear need for novel, more effective therapies to prevent relapse, control metastases and improve overall survival. Improved understanding of SCCHN disease biology has led to the introduction of molecularly targeted treatment strategies in these cancers. The epidermal growth factor receptor (EGFR) is expressed at much higher levels in SCCHN tumours than in normal epithelial tissue, and EGFR expression correlates with poor prognosis. Therefore, much effort is currently directed toward targeting aberrant EGFR activity (e.g. cell signalling) in SCCHN. This review discusses the efficacy of novel therapies targeting the EGFR (e.g. anti-EGFR antibodies and EGFR tyrosine kinase inhibitors) that are currently tested in SCCHN patients.

Head and neck cancer (HNC) accounts for about 5% of all cancers with >500 000 cases diagnosed worldwide and >100 000 in Europe each year. The majority of HNC in the Western world is of squamous cell origin (>90% squamous cell carcinoma of the head and neck, SCCHN) and present with locally or regionally advanced disease (Rogers *et al*, 2005). Patients with early-stage disease are treated with surgery and/or radiotherapy and nearly 80% are cured. Chemotherapy added to locoregional treatment provides a demonstrated survival benefit in nonmetastatic SCCHN (Pignon *et al*, 2000). However, despite combined treatment approaches (surgery and radiation/chemoradiation therapy) most patients with resectable advanced disease develop local or regional recurrences (50–60%), metastatic disease (∼20%) or secondary primaries. Patients with unresectable advanced disease have a 5-year survival of <10% and recurrent/metastatic cases have a median survival of approximately 6–9 months, which has not changed significantly for 30 years. Several therapeutic options are available for these patients, including (re-)irradiation, salvage surgery, palliative chemotherapy or best supportive care for patients with low performance status. The most commonly used agents are cisplatin or carboplatin, often in combination with taxanes or 5-fluorouracil. Response rates (RR) to first-line platinum-based chemotherapy are only ∼30%. In recurrent/metastatic SCCHN, survival benefits of 10 weeks may be expected (Morton *et al*, 1985; Browman and Cronin, 1994). Although several combinations of classical chemotherapeutics have increased RR, improved survival has not been observed. Options and RR of patients refractory to platinum-based therapies are generally very poor. Therefore, there is clearly an unmet therapeutic need for new active, less toxic agents for SCCHN treatment.

## TARGETING EPIDERMAL GROWTH FACTOR- RECEPTOR SIGNALLING IN SCCHN

Several molecularly targeted strategies have been evaluated in HNC patients owing to (1) mechanism of action, (2) greater selectivity and (3) different/lower toxicity. Potential targets are growth factor receptors, signal transduction, cell cycle control, protein degradation, hypoxia, angiogenesis and prostaglandin synthesis. The epidermal growth factor-receptor (EGFR) is a particularly interesting target as it plays an important role in regulation of cellular proliferation, differentiation and survival of epithelial cells and tumours of epithelial cell origin. Additionally, aberrant EGFR signalling imparts SCCHN cells with classic tumour cell characteristics, including decreased apoptosis, enhanced invasiveness, migration, angiogenesis and metastasis. Furthermore, EGFR is overexpressed in approximately 90–100% of SCCHN specimens and has been associated with worse prognosis, including advanced stage, poorly differentiated tumours and poor survival (Santini *et al* 1991; Dassonville *et al*, 1993). Epidermal growth factor-receptor is one of four transmembrane growth factor receptors that share structural and functional similarities, including EGFR (=HER1, c-erbB-1), HER-2/neu (c-erbB-2), HER3 (c-erbB-3) and HER4 (c-erbB-4). The EGFR, a 170 kDa glycoprotein, consisting of an extracellular domain, a transmembrane region and an intracellular domain with tyrosine kinase function, responds to numerous ligands, such as transforming growth factor alpha (TGF-*α*), betacellulin, amphiregulin, epiregulin, EGF and heparin-binding EGF (Rogers *et al*, 2005).

**Figure 1:**
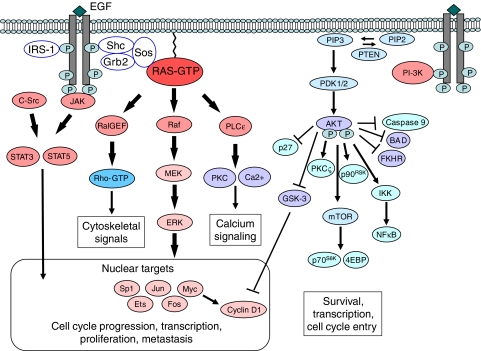
Intracellular signalling of the EGFR. Shown are the major signalling pathways downstream of c-erbB-receptors (e.g. EGFR). Modified after Rogers *et al* (2005) and Kalyankrishna and Grandis (2006). Binding of specific ligands (e.g. EGF, heparin-binding EGF, TGF-*α*, amphiregulin, betacellulin and heregulin) may generate up to 10 types of homo- or heterodimeric complexes resulting in conformational changes in the intracellular EGFR kinase domain, which lead to autophosphorylation and activation. Consequently, signalling molecules, including growth factor receptor-bound protein-2 (Grb-2), Shc and IRS-1 are recruited to the plasma membrane. G-protein coupled receptors can also activate EGFR in a ligand-independent manner by Src-mediated direct phosphorylation of Y-845. Insulin-like growth factor-1 receptor can also transactivate the EGFR. Activation of several signalling cascades is triggered predominately by the RAS-to-MAPK and the PI-3K/Akt pathways, resulting in enhanced tumour growth, survival, invasion and metastasis.

Downstream effects of EGFR activation after receptor dimerisation, internalisation and autophosphorylation are mediated through several signal transduction pathways involving the RAS/MAP kinase, the phosphatidylinositol 3-kinase (PI-3K)/Akt, the PLC*γ* and the JAK-STAT pathways (Rogers *et al*, 2005; Kalyankrishna and Grandis, 2006) (Figure 1). Although the main autophosphorylation sites in ErbB receptors recruit extensively overlapping molecules to the active receptors, preferential modulation of signalling pathways seems to occur (e.g. EGFRs with kinase-domain mutations preferentially activate the pro-survival PI-3K/AKT pathway and the STAT pathway). Downstream effectors of EGFR (e.g. ERK-1/2, AKT, STAT-3/5) are activated in SCCHN (Kalyankrishna and Grandis, 2006). Epidermal growth factor receptor ligand binding results in several homo- or heterodimeric complexes. Furthermore, EGFR can be activated by other receptor tyrosine kinases including insulin-like growth factor-1 receptor, adhesion molecules (e.g. E-cadherin and integrins) and G-protein-coupled receptors (GPCR).

Deregulation of EGFR function is a common feature in several human malignancies including lung, breast, colorectal, prostate and HNC. Mechanisms of EGFR activation include (1) receptor overexpression in most epithelial malignancies (EGFR in up to 90% of SCCHN; ErbB2 in 3–29%; ErbB3 in 21% and ErbB4 in 26%), (2) constitutively activated EGFR mutants, (3) autocrine activation by ligand overexpression (e.g. TGF-*α*), (4) ligand-independent activation through other receptor systems (e.g. ErbB2/HER2), (5) EGFR transactivation by GPCR-induced processing of transmembrane growth factor precursors by ADAM family metalloproteases, (6) gene amplification and/or (7) loss of negative regulatory mechanisms (Rogers *et al*, 2005; Kalyankrishna and Grandis, 2006).

Dysregulated p53, polymorphisms in dinucleotide repeats in intron 1 of the EGFR gene and EGFR amplification can all lead to increased EGFR mRNA synthesis. However, EGFR gene amplification was only observed in seven out of 33 patients with SCCHN and did not correlate with EGFR protein overexpression, suggesting that gene amplification is not pathogenetically involved in EFGR protein overexpression (Mrhalova *et al*, 2005). Furthermore, overexpression of cortactin may inhibit ligand-induced EGFR downregulation. Interestingly, tobacco smoke increases EGFR ligand levels (e.g. amphiregulin, TGF-*α*) culminating in EGFR activation and increased levels of cyclooxygenase 2 and prostaglandin E2, which can transactivate EGFR (Kalyankrishna and Grandis, 2006).

Recently, three identical in-frame deletions in exon 19 (E746_A750del) of the EGFR gene were reported in three out of 41 (7.3%) Korean SCCHN cases (Lee *et al*, 2005). In contrast, EGFR kinase domain mutations were rare among US (zero out of 65) or European (one out of 100) SCCHN cases (Cohen *et al*, 2005a; Loeffler-Ragg *et al*, 2006). Interestingly, one gefitinib-responsive SCCHN patient harboured a heterozygous mutation within ErbB2 (V773A) (Cohen *et al*, 2005a). ErbB2 heterodimerises with EGFR and ErbB2 mutations have recently been reported within a subset of non-small cell lung cancer (NSCLC). Epidermal growth factor receptor vIII, a deletion of exons 2–7 resulting in a truncated extracellular domain and constitutive tyrosine kinase activation, has been reported in glioblastoma multiforme (>50%; Moscatello *et al*, 1995), NSCLC (1–42%; Ji *et al*, 2006; Sonnweber *et al*, 2006) and in SCCHN (42%; Sok *et al*, 2006).

### Inhibition of EGFR signalling

**Figure 2:**
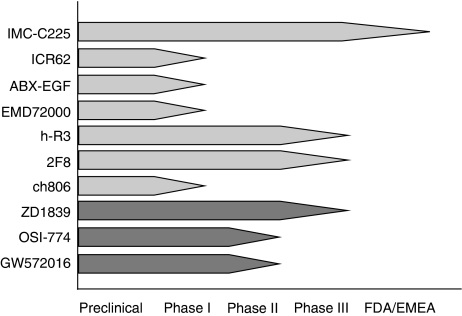
Preclinical and clinical development of Mabs and TKIs targeting the EGFR in SCCHN. The Mabs (light grey arrows), tested to date, include IMC-C225 (cetuximab), ICR62, ABX-EGF (panitumumab), EMD72000 (matuzumab), h-R3 (nimotuzumab), 2F8 (zalutumumab) and ch806. Cetuximab (IMC-C225) has been approved for use in SCCHN by both the FDA and EMEA in combination with radiotherapy. Nimotuzumab (h-R3) was recently approved for head and neck cancer in Argentina, Cuba, Columbia, China and India. ch806 is an EGFRvIII-specific Mab. Tyrosine kinase inhibitors (dark grey arrows) in clinical development include the EGFR inhibitors ZD1839 (gefitinib) and OSI-774 (erlotinib; formerly known as CP-358-774) as well as the EGFR/HER-2 inhibitor GW572016 (lapatinib).

Several approaches to block EGFR signalling in human diseases have been tested, including (1) monoclonal antibodies (Mabs), (2) small molecule tyrosine kinase inhibitors (TKIs), (3) inhibition of receptor trafficking to the cell membrane and (4) inhibition of EGFR synthesis through antisense oligonucleotides. Only Mabs against EGFR and EGFR-specific TKIs have been evaluated in phase III trials (Figure 2). Inhibition of EGFR signalling has been used in primary treatment of locally advanced SCCHN with radiation therapy and as first/second-line agents in recurrent/metastatic SCCHN.

### Anti-EGFR Mabs

EGFR-specific Mabs and their characteristics are listed in Table 1. Mechanisms of action include (1) inhibition of receptor activation and signalling by blocking ligand binding to the extracellular domain, and (2) induction of antibody-dependent cell-mediated cytotoxicity and/or complement-dependent cytotoxicity.

#### Anti-EGFR Mabs in recurrent/metastatic SCCHN

Cetuximab (IMC-C225) has antitumour activity against several tumour cell lines expressing EGFR and in SCC tumour xenograft models. Cetuximab has been studied in several phase I–III trials in locoregionally advanced or recurrent/metastatic SCCHN patients (Table 2). In three phase I trials (including 26 SCCHN patients) of cetuximab as a single, weekly multiple dose (5–400 mg m^−2^) with or without cisplatin (60 mg m^−2^ once every 4 weeks), disease stabilisation was observed without reaching MTD. Nine out of 13 patients treated with cetuximab doses ⩾50 mg m^−2^ plus cisplatin completed 12 weeks of therapy including two partial responses (PRs) in SCCHN after treatment with 200 and 400 mg m^−2^ (Baselga *et al*, 2000). Another phase Ib study combining cetuximab with cisplatin in recurrent SCCHN reported two complete remissions (CRs) and four PRs of nine patients (Shin *et al*, 2001). The most frequently occurring adverse events (AEs) were fever/chills, asthenia, transaminase elevation, nausea and skin toxicities including acneiform rashes, flushing and seborrheic dermatitis. Grade 3–4 AEs included aseptic meningitis, allergic reaction, epiglottitis plus dyspnoe.

Antitumour activity of cetuximab plus cisplatin in platinum-refractory SCCHN patients was recently reported. In a multicentre phase II trial, 132 SCCHN patients were treated with two 3-week cycles of cisplatin/paclitaxel or cisplatin/5-fluorouracil (Herbst *et al*, 2005). Patients with a CR or PR continued standard therapy. Patients with stable disease (SD; *n*=51) or progressive disease (PD/1; *n*=25) received cetuximab plus cisplatin (75 or 100 mg m^−2^ every 3 weeks). Patients who developed PD within 90 days (PD/2; *n*=54) were subsequently enrolled to cetuximab plus cisplatin. Objective responses were observed in 5, 3 and 9 patients with a median response duration of 4.2, 4.1 and 7.4 months and a median overall survival (OS) of 6.1, 4.3 and 11.7 months for the PD/1, PD/2 and SD groups, respectively. The most common toxicities were anaemia, acne-like skin rash, leukopenia, fatigue/malaise and nausea/vomiting. Seven patients developed grade 3/4 hypersensitivity reactions to cetuximab (Herbst *et al*, 2005).

Baselga *et al* (2005) reported another multicentre phase II trial with 96 platinum-refractory SCCHN patients who received cetuximab plus cisplatin (⩾60 mg m^−2^ cycle^−1^) or carboplatin (⩾250 mg m^−2^ cycle^−1^). In the intent-to-treat population, the RR was 10% with a disease control rate (DCR=CR+PR+SD) of 53%. The median time to progression (TTP) was 85 days and OS 183 days, respectively. Treatment was well tolerated with skin reactions being the most common cetuximab-related event.

Cetuximab also exhibited single-agent activity in platinum-refractory SCCHN patients. In a multicentre phase II study with 103 evaluable patients, a 16.5% RR was reported. Median TTP and OS were 85 and 175 days, respectively (Trigo *et al*, 2004).

In a phase III randomised multicentre placebo-controlled ECOG trial in 117 metastatic/recurrent SCCHN patients, cetuximab plus cisplatin (100 mg m^−2^ every 4 weeks) was compared with cisplatin plus placebo (Burtness *et al*, 2005). The hazard ratio (HR) for progression (primary end point) for the combination compared to cisplatin plus placebo was 0.78 (95% CI, 0.54–1.12) with a median PFS of 4.2 *vs* 2.7 months (*P*=0.09), respectively. This was not significant as the study was powered to detect a 50% reduction in HRs. Furthermore, both arms had a significant drop-off rate and the control arm performed better than expected. Median OS was 9.2 months for cisplatin plus cetuximab and 8.0 months for cisplatin plus placebo (*P*=0.21) and the objective RR was 26 *vs* 10%, respectively (*P*=0.03). However, there was a survival advantage for the development of rash (HR for survival by skin toxicity in cetuximab-treated patients 0.42 (95% CI, 0.21–0.86)).

Zalutumumab (HuMax-EGFr^R^, 2F8) was recently tested in a phase I/II trial in 24 patients with recurrent/metastatic SCCHN (0.15–8 mg kg^−1^ iv, after 28 days 4 × weekly). Two PRs and eight SDs were observed in 15 evaluable patients all occurring in the 1, 2, 4 and 8 mg kg^−1^ dose group (Bastholt *et al*, 2005). In January 2006, FDA awarded Fast Track status to zalutumumab for HNC patients who previously failed standard therapies. A pivotal phase III study with zalutumumab in 273 SCCHN patients who are refractory to or intolerant of standard platinum-based chemotherapy was initiated in September 2006.

#### Anti-EGFR-Mabs in combination with radiotherapy

In a phase I study in locoregionally advanced SCCHN patients, cetuximab was delivered in combination with conventional or hyperfractionated RT (Robert *et al*, 2001). All patients achieved an objective response (13 CRs and two PRs). The recommended dose for phase II/III trials was 400–500 mg m^−2^ loading dose and 250 mg m^−2^ weekly maintenance dose. A pilot phase II study of concurrent cetuximab, cisplatin and concomitant boost radiotherapy for locoregionally advanced SCCHN patients (*n*=32) reported a 3-year OS of 75%, PFS of 56% and a locoregional control rate of 71%. However, the study was closed for significant AEs including two deaths (Pfister *et al*, 2006). A randomised phase III study compared radiation with or without cetuximab for patients with locally advanced inoperable SCCHN patients (Bonner *et al*, 2006). The median duration of locoregional control was 24.4 months among patients treated with cetuximab plus radiotherapy and 14.9 months among those given radiotherapy alone (HR for locoregional progression or death, 0.68; *P*=0.005). With a median follow-up of 54.0 months, the median OS was 49.0 months among patients treated with combined therapy and 29.3 months among those treated with radiotherapy alone (HR for death, 0.74; *P*=0.03). Radiotherapy plus cetuximab significantly prolonged PFS (HR for disease progression or death, 0.70; *P*=0.006). Cetuximab did not significantly add to the acute side effects of radiotherapy, offering a real therapeutic advantage to patients who are ineligible to receive standard chemoradiation. As a result of this study, cetuximab was approved by the FDA/EMEA in combination with radiotherapy to treat SCCHN in February and April 2006. However, this study did not compare cetuximab plus radiotherapy with platinum-based radiotherapy, which is the current standard of care. Additionally, radiotherapy was not uniformly administered among all patients. These shortcomings are currently addressed in RTOG trial 0522, which has been recently initiated and compares chemoradiation with cisplatin to chemoradiation plus cetuximab.

Nimotuzumab (h-R3) has demonstrated clinical benefit without rash development in several clinical trials. In a single-centre phase I/II trial with 24 locally advanced SCCHN patients who received 6 × weekly infusions (cumulative doses of 300, 600, 1200 and 2400 mg) plus radiotherapy (60–66 Gy), the combination was well-tolerated with no skin or allergic toxicity (Crombet *et al*, 2004). Nimotuzumab was recently approved for nasopharyngeal cancer in China (April 2005), based on a 75% improvement in CR (91 *vs* 52%) in a phase II trial in 130 patients diagnosed with squamous cell nasopharyngeal carcinoma who were treated with nimotuzumab plus radiotherapy *vs* radiotherapy alone. It has also been approved for the treatment of HNC in Argentina, Columbia, Cuba and India (July 2006). A phase III trial in HNC is currently ongoing.

### EGFR TKIs

Numerous protein kinase inhibitors have been developed including inhibitors of the EGFR kinase domain. Some molecules are highly specific for EGFR (e.g. ZD1839, OSI-774), while others may block additional Erb family kinases (e.g. GW572016, PKI-66) or other protein kinase families (ZD6474). Both ZD1839 (Gefitinib) and OSI-774 (formerly known as CP-358-774, Erlotinib) have FDA approval for treatment of locally advanced or metastatic NSCLC since May 2003 and November 2004, respectively. Three orally active EGFR inhibitors have been tested in clinical trials in recurrent/metastatic SCCHN or in combination with radiotherapy in locoregionally advanced SCCHN (Table 3).

### TKIs in recurrent/metastatic SCCHN

Gefitinib (Iressa^R^, AstraZeneca Pharmaceuticals, London, UK) impeded *in vitro* and *in vivo* growth of cell lines that express high, intermediate or low levels of EGFR and high levels of HER-2. Furthermore, gefitinib has additive or synergistic properties in combination with cisplatin, carboplatin, paclitaxel, taxanes, doxorubicin and radiotherapy. A phase II trial of 500 mg gefitinib in 52 patients with recurrent/metastatic SCCHN reported an RR of 10.6% and a DCR of 53% (Table 3) (Cohen *et al*, 2003). Half the cohort received gefitinib as second-line therapy. Median TTP and OS were 3.4 and 8.1 months, respectively. The only grade 3 toxicity encountered was diarrhoea (*n*=3). Performance status and development of skin toxicity were found to be strong predictors of response, progression and survival. In another phase II trial, gefitinib (500 mg dose^−1^, reduction to 250 mg) was tested in 32 patients with recurrent SCCHN. In cohort A (no chemotherapy), three PR and six SD were observed out of 20 patients (clinical benefit in 45%). In cohort B (one previous chemotherapy), three out of 12 patients achieved SD (25% clinical benefit). There was no association between rash incidence/grade and clinical benefit (Wheeler *et al*, 2005). An expanded access program with gefitinib (500 mg day^−1^) in 47 SCCHN patients reported an 8% clinical RR with 36% DCR. The median TTP and OS were 2.6 and 4.3 months, respectively. Acneiform folliculitis was the most frequent toxicity observed (76%) (Kirby *et al*, 2006). In another phase II trial with gefitinib (250 mg day^−1^) in 70 SCCHN patients, two PRs and a 34% DCR were observed. Median TTP and OS were 1.8 and 5.5 months, respectively, with no difference between untreated and pretreated patients. Gefitinib monotherapy at 250 mg day^−1^ in recurrent/metastatic SCCHN may have less activity than was previously observed for 500 mg daily. Squamous cell carcinomas of the head and neck responses to gefitinib or erlotinib seem not to be linked to EGFR kinase mutational status, as these mutations are rare in this disease (Cohen *et al*, 2005a, 2005b). Recently, a phase I study in SCCHN reported that gefitinib (250/500 mg q.d.) in combination with celecoxib (200/400 mg b.i.d.) is very well tolerated in patients with incurable SCCHN (Wirth *et al*, 2005).

Erlotinib (Tarceva^R^; Roche, Genentech, OSI Pharmaceuticals) has FDA/EMEA approval as single-agent treatment for patients with locally advanced or metastatic NSCLC, and FDA approval in combination with gemcitabine for first-line treatment of patients with locally advanced, unresectable or metastatic pancreatic cancer. A multicentre phase II trial of erlotinib in 115 patients with recurrent and metastatic SCCHN reported a 4.3% overall RR, including five PRs (Soulieres *et al*, 2004). Disease stabilisation was maintained in 44 patients (38.3%) for a median duration of 16.1 weeks. Median PFS was 9.6 weeks and median OS was 6.0 months. Rash (79%) and diarrhoea (37%) were the most common drug-related toxicities. Skin rashes (>grade 2) correlated with longer OS (7.4 months *vs* 4.0 (grade 0) or 5.0 months (grade 1)). Recently, a phase I study in SCCHN reported that erlotinib (150 mg qd) in combination with bevacizumab (5, 10 and 15 mg kg^−1^ i.v. 3q weeks) is feasible and well tolerated in patients with recurrent/metastatic SCCHN (Mauer *et al*, 2004). In a phase II trial of erlotinib (150 mg p.o.) plus cisplatin/docetaxel in recurrent/metastatic SCCHN, 66% ORR (three CRs, 18 PRs, eight SDs) and 91% DCR were observed. Grade 3/4 toxicities included neutropenia, diarrhoea and rash (Kim *et al*, 2006).

Lapatinib (Tykerb^R^, GW572016, GlaxoSmithKline, Brentford, London, UK) is a selective kinase inhibitor of both EGFR and HER-2. A phase II trial of lapatinib (1500 mg o.d.) in recurrent/metastastic SCCHN reported no objective responses, suggesting little activity in either EGFR inhibitor naïve or refractory patients (Abidoye *et al*, 2006) (Table 3).

### TKIs in combination with radiotherapy

A phase I/II study combining gefitinib (250 mg p.o. q.d.) with induction chemotherapy (docetaxel/5-FU/carboplatin) in locally advanced SCCHN demonstrated that this regimen was feasible and produced high RR (11 CRs, 18 PRs, five SDs out of 34 patients) (Doss *et al*, 2006). Two phase I/II studies of erlotinib in combination with docetaxel or cisplatin and radiotherapy in locally advanced SCCHN demonstrated that these combinations are safe and feasible (Herchenhorn *et al*, 2006; Savvides *et al*, 2006). Phase II trials are planned or ongoing (Table 4). Recently, results from an ongoing phase I study of lapatinib in combination with cisplatin (100 mg m^−2^ days 1, 22, 43) plus radiotherapy (66–70 Gy/6–7 weeks) in locally advanced SCCHN demonstrated minor AEs and encouraging clinical activity (Harrington *et al*, 2006).

### Rash and clinical outcome

Acneiform papulopustular skin rash, usually on the face and upper torso, is the most common toxicity found with EGFR antibodies such as cetuximab, panitumumab or matuzumab and the kinase inhibitors gefitinib and erlotinib (Perez-Soler and Saltz, 2005). Rash was mainly grade 1/2, with grade 3 in <13% of patients and no grade 4. Phase I studies indicate that rash is dose dependent. Data from multiple studies with cetuximab, erlotinib and gefitinib show a consistent relationship between rash and response or survival (Cohen *et al*, 2003, 2005a, 2005b; Soulieres *et al*, 2004; Baselga *et al*, 2005; Burtness *et al*, 2005; Herbst *et al*, 2005). Little is known about the aetiology of the rash and evidence-based treatment recommendations for rash management are missing owing to the lack of clinical trials addressing this problem (Perez-Soler and Saltz, 2005). Recently, results from a prospective algorithmic approach for the treatment of skin rash in SCCHN patients was presented (Garey *et al*, 2005) (Table 5). Responses included 11 out of 11 CRs for grade 1, three out of four PRs for grade 2 and one PR for grade 3 rash.

## CONCLUSIONS AND FUTURE PERSPECTIVES

Clinical trials treating SCCHN patients with molecularly targeted treatment strategies designed to specifically inhibit EGFR function have shown promising – albeit limited – levels of efficacy, even as monotherapy. Food and Drug Administration/EMEA approval of cetuximab, in combination with radiotherapy for SCCHN treatment, represents the first new drug registration to treat HNC since methotrexate became available in the 1950s. Cetuximab or TKIs plus radiotherapy have a more favourable toxicity profile when compared to chemoradiotherapy. Multiple phase I/II trials are currently testing combinations of cetuximab or TKIs with chemoradiotherapy in locoregionally advanced SCCHN (Table 4). In recurrent/metastatic SCCHN, cetuximab and three TKIs are currently investigated as monotherapy or in combination with chemotherapy. Owing to high toxicity with cisplatin combinations, taxane regimens may be more feasible. These combination therapies may lead to the use of lower doses of standard chemotherapeutics and thus reduced non-specific toxicity to patients, without loss of anticancer activity. Cisplatin-refractory, recurrent/metastatic SCCHN patients may also benefit from EGFR-targeting strategies.

Interestingly, dual-agent molecular targeting of the EGFR combining cetuximab with TKIs (e.g. gefitinib, erlotinib) enhanced tumour growth inhibition over that observed with either agent alone (Huang *et al*, 2004; Matar *et al*, 2004). However, others have found that combination of cetuximab and gefitinib was antagonistic in all cell lines considered, suggesting that a double-hit strategy with Mabs and TKIs must be considered with caution (Fischel *et al*, 2005). Another strategy may be combination of EGFR antagonists (Mab or TKI) with inhibitors of RAS or phosphatidyl inositol-3 kinase pathways, which are downstream of the EGFR (e.g. PI3K inhibitor LY294002, MEK inhibitor U0126, mevalonate pathway inhibitor lovastatin or farnesyl transferase inhibitor FTI SCH66336). Furthermore, inhibitors of VEGF signalling (e.g. bevacizumab, sorafenib, AZD2171) may be potent partners for novel combination therapies (Table 4). Other potentially interesting targets are EGFR-independent survival pathways (e.g. insulin-like growth factor-1 (IGF-1R)) or GPCRs that mediate their effects through EGFR.

Although anti-EGFR-targeted therapies may lead to PRs and disease stabilisation in some patients, many patients do not benefit from these therapies and responsive cases may eventually develop resistance. Molecular resistance mechanisms include (1) specific EGFR mutations (e.g. EGFRvIII, T790M), (2) constitutive activation of downstream effectors (e.g. loss/inactivation of PTEN, activation of Src, RAS, STAT3/5), (3) increased angiogenesis (upregulation of VEGF) and (4) the presence of redundant tyrosine kinase receptors (e.g. HER-2, c-MET, IGF-1R) (Kobayashi *et al*, 2005). Many anti-EGFR Mabs are unable to bind the aberrant extracellular domain of EGFRvIII and thus fail to inhibit ligand-induced receptor activation. Interestingly, resistance caused by the T790M mutation can be overcome by CL-387785, a specific and irreversible anilinoquinazoline EGFR inhibitor (Kobayashi *et al*, 2005). Another major challenge is the development of reliable methods to determine which patient populations are likely to receive the greatest benefit from these novel agents in order to justify the enormous treatment costs of these new drugs.

## Figures and Tables

**Table 1:** Anti-EGFR Mab

**Product**	**Type**	**Ig subclass**	**Affinity (M)**	**EGFR Epitope**	**Rash**	**Indications**	**Clinical development**	**Company**
Cetuximab (IMC–C225) (Erbitux^R^)	Chimeric murine Mab225	IgG_1_	3.9 × 10^−10^	Domain III	+++	HNC, CRC	FDA/EMEA	ImClone, Merck, BMS
Nimotuzumab (h–R3) (TheraCIM h–R3^R^ in North America) (Theraloc^R^ in Europe) (CIMAher in Latin America)	Humanised Mab egf/r3	IgG_1_	10^−9^–10^−10^	Region 400–410 3A	^−^	HNC, glioma	Phase III	Oncoscience, Biotech Pharma, YM Biosciences, Biocon, CIMAB SA
Zalutumumab (2F8) (HuMax–EGFr^R^)	Human	IgG_1_			+++	HNC	Phase III	Genmab A/S, Medarex
Matuzumab (EMD72000)	Humanised EMD55900 Mab425	IgG_1_	3.4 × 10^−10^		+++	Gastric, NSCLC	Phase II	Merck, Takeda
Panitumumab (ABX–EGF)	Human	IgG_2_	5 × 10^−11^		+++	CRC, NSCLC	Phase III	Abgenix, Amgen
ICR62	Rat	IgG_2b_		Epitope C			Phase I	The Institute ofCancer Research (UK)
Ch806	Humanised murine Mab806	IgG_1_	1.1 × 10^−9^	Region 287–302 EGFRvIII			Phase I	Ludwig Institute for Cancer Research (Melbourne)

CRC=colorectal cancer; EGFR=epidermal growth factor receptor; HNC=head and neck cancer; Mab, monoclonal antibody; NSCLC=non-small cell lung cancer.

Cetuximab has EMEA/FDA approval for treatment of metastatic CRC and was recently approved in combination with radiotherapy for the treatment of SCCHN. Nimotuzumab was recently approved in combination with radiotherapy for nasopharyngeal cancer in China. It has also been approved for the treatment of HNC in Argentina, Columbia, Cuba and India (July 2006).

**Table 2:** Clinical trials of anti-EGFR antibodies for therapy of SCCHN

**Drug**	**Phase**	**N**	**Dosage**	**Stage**	**Response rate**	**Reference**
*Anti-EGFR antibodies in combination with chemotherapy or as monotherapy:*						
Cetuximab (IMC–C225)	I	26	5–100 (200–400) mg m^−2^ SiD, MuD, combination+cisplatin 60 mg m^−2^ 4w^−1^	Advanced	PR^a^ 2	Baselga *et al* (2000)
Cetuximab (IMC–C225)	Ib	12	100–500 mg m^−2^ LD 100–250 mg m^−2^ MD weekly 6w+cisplatin 100 mg m^−2^ 3w^−1^	Recurrent	OR^a^ 67% (6/9) CR^a^ 2 PR^a^ 4	Shin *et al* 2001)
Cetuximab (IMC–C225)	II	132	400 mg m^−2^ LD 250 mg m^−2^ MD weekly 4 × +cisplatin 75/100 mg m^−2^ 3w^−1^	Recurrent, P-refractory	OR^a^ 13% (17/130) CR^a^ 2 PR^a^ 15 SD^a^ 66 DCR^a^ 64%	Herbst *et al* (2005)
Cetuximab (IMC–C225)	II	96	400 mg m^−2^ LD 250 mg m^−2^ MD weekly+cisplatin/Carboplatin	Recurrent, P-refractory	OR^a^ 10% CR^a^ 0 DCR^a^ 53%	Baselga *et al* (2005)
Cetuximab (IMC–C225)	II	103	400 mg m^−2^ LD 250 mg m^−2^ MD weekly	Recurrent, P-refractory	OR^a^ 16.5% CR^a^ 5 PR^a^ 12 SD^a^38 DCR^a^ 53.4%	Trigo *et al* (2004)
Cetuximab (IMC–C225)	III	117	A: C225+P B: placebo+P	Recurrent/metastatic	OR^b^ A26%/B10% (p=0.03) OS A 9.2/B 8.0 m (n.s.)	Burtness *et al* (2005)
Zalutumumab (2F8)	I–II	24	0.15–8 mg kg^−1^ d28 weekly	Recurrent	OR^b^ 12.5% PR^b^ 2 SD^b^ 8	Bastholt *et al* (2005)
						
*Anti–EGFR antibodies in combination with radiotherapy:*						
Cetuximab (IMC–C225)	I	16	100–500 mg m^−2^ LD 100–250 mg m^−2^ MD for 7–8 weeks+RT (70 Gy, 2 Gy/d or 76.8 Gy, 1.2 Gy b.i.d.)	Advanced untreated	OR^a^ 100% CR^a^ 13 PR^a^ 2	Robert *et al* (2001)
Cetuximab (IMC–C225)	II	22	400 mg m^−2^ LD 250 mg m^−2^ MD weekly+boost radiotherapy (70 Gy)+cisplatin (100 mg m^−2^ w1+4)	Locoregionally advanced	OR^a^ 15/16 CR^a^ 2 PR^a^ 13 OS (3y) 76% PFS (3y) 56%, LCR 71%	Pfister *et al* (2006)
Cetuximab (IMC–C225)	III	424	A : radiotherapy B : radiotherapy+cetuximab 400 mg m^−2^ LD, 250 mg m^−2^ MD	Locoregionally advanced	A : OS 29.3 mo. B : OS 49 mo.	Bonner *et al* (2006)
Nimotuzumab (h–R3)	I	17	50–400 mg weekly 6w+RT (60–66 Gy; 2 Gyd^−1^)	Advanced	OR^b^ 87.5% (14/16) CR^b^ 9	Crombet *et al* (2004)

d=day; CR=complete remission; EGFR=epidermal growth factor receptor; DCR=disease control rate; LD=loading dose; MD=maintenance dose; mo.=months; MuD=multiple doses; OR=overall response rate; OS=median overall survival; PFS=median progression-free survival; PR=partial remission; RT=radiotherapy; SCCHN=squamous cell carcinomas of the head and neck; SD=stable disease; SiD=single dose; TTP=median time to progression; w=week; y=year.

a WHO criteria;

b RECIST=response evaluation criteria in solid tumours.

**Table 3:** Clinical trials of EGFR tyrosine kinase inhibitors for therapy of SCCHN

**Drug**	**Phase**	**N**	**Dosage**	**Stage**	**Response rate**	**Reference**
*EGFR TKIs given as monotherapy or in combination with chemotherapy:*						
Gefitinib	II	52	500 mg day^−1^	Rec./met.	OR^b^ 10.6%, DCR^b^ 53% CR^b^ 1 PR^b^ 4 SD^b^ 20 TTP 3.4 mo., OS 8.1 mo.	Cohen *et al* (2003)
Gefitinib	II	32	250–500 mg day^−1^ A: no prior chemotherapy B: one prior chemotherapy	Rec.	OR^b^ 9.4% A: PR^b^ 3 SD^b^ 6 (20) B: SD^b^ 3 (12) TTP 3 mo., OS 6 mo.	Wheeler *et al* (2005)
Gefitinib	ea	47	500 mg day^−1^	Rec./met.	OR^b^ 8%, DCR^b^ 36% PR^b^ 4 SD^b^ 13 TTP 2.6 mo., OS 4.3 mo.	Kirby *et al* (2006)
Gefitinib	II	70	250 mg day^−1^	Rec./met.	OR^b^ 1.4%, DCR^b^ 34% PR^b^ 1 SD^b^ 23	Cohen *et al* (2005b)
					TTP 1.8 mo. OS 5.5 mo.	
Erlotinib	II	115	150 mg day^−1^	Rec./met.	OR^a^ 4.3%, DCR^a^ 38.3% PR^a^ 5 SD^a^ 44 PFS 9.6 w, OS 6.0 mo.	Soulieres *et al* (2004)
Erlotinib+cisplatin, docetaxel	II	37	150 mg day^−1^	Rec./met.	OR^b^ 66% (21/32), DCR^b^ 91% CR^b^ 3 PR^b^ 18 SD^b^ 8	Kim *et al* (2006)
Lapatinib	II	42	1500 mg day^−1^	Rec./met. A: naïve B: TKI pre-treated	OR 0% SD A:37%; B:20% PFS A:1.6 mo.; B:1.7 mo.	Abidoye *et al* (2006)
						
*EGFR TKIs in combination with radiotherapy:*						
Gefitinib+induction chemotherapy followed by radiochemotherapy	II	45	250 mg qd^−1^	Locally-advanced unresectable	OR^a^ 85% (29/34) CR^a^ 11 PR^a^ 18 PFS 1.8 mo., OS 5.5 mo.	Doss *et al* (2006)
Erlotinib+radiochemotherapy (docetaxel)	I	23	15 mg m^−2^ (50 mg day^−1^) 15 mg m^−2^ (100 mg day^−1^) 20 mg m^−2^ (100 mg−day^−1^) 20 mg m^−2^ (150 mg day^−1^)	Locally-advanced		Savvides *et al* (2006)
Lapatinib+radiochemotherapy	I	17	500–1500 mg day^−1^	Locally-advanced		Harrington *et al* (2006)

CR=complete remission; DCR=disease control rate (CR+PR+SD); ea=expanded access programme; EGFR=epidermal growth factor receptor; met.=metastatic; OR=overall response rate (CR+PD); OS=median overall survival; P=cisplatinum; PFS=median progression free survival; PR=partial remission; rec.=recurrent; SCCHN=squamous cell carcinomas of the head and neck; SD=stable disease; TTP=median time to progression.

a WHO criteria;

b RECIST=response evaluation criteria in solid tumours.

**Table 4:** Ongoing clinical trials targeting the EGFR in SCCHN (www.clinicaltrials.com)

**Drug**	**Phase**	**SCCHN/Stage**	**N**	**Sponsor**
*TKIs as monotherapy or in combination with chemotherapy:*				
Erlotinib	II	Recurrent/metastatic	37	NCI
Erlotinib+docetaxel	I/II	Recurrent/metastatic	15/36	Ohio State Univ. NCI
Erlotinib±bevacizumab	I/II	Advanced	30	Duke Univ. Genentech./OSI
Erlotinib+docetaxel+cisplatin	II	Recurrent/metastatic	50	MD Anderson CC
Docetaxel±gefitinib	III	Recurrent/metastatic	330	ECOG
Lapatinib	II	Recurrent/metastatic	15–30	Univ. Virginia NCI
Lapatinib	II	Recurrent/metastatic	40–88	Univ. Chicago NCI
				
*TKIs in combination with radiotherapy:*				
Erlotinib+cisplatin ia+RTX	II	Locally-advanced	20	Southern Illinios Univ. Genentech./OSI
Erlotinib+RTX±cisplatin	I	Stage II–IV	24–48	Sidney Kimmel CC NCI
Erlotinib+docetaxel+RTX	I	Locoregionally advanced	24	MD Anderson CC Sanofi-Aventis Genentech
Adjuvant Erlotinib after RCTX	I	Locally-advanced	6–20	NCI Canada
Gefitinib +RTX	II	Locally-advanced inoperable	28	AstraZeneca
Gefitinib+cisplatin+RTX	I/II	Locally-advanced	40	AstraZeneca
Gefitinib+cisplatin+Re-RTX	I	Locoregional recurrent	10	Stanford Univ. AstraZeneca
Gefitinib+cisplatin+RTX0	I/II	Unresectable	29	Cornell Univ.
Gefitinib+Paclitaxel+RTX	I	Advanced/recurrent	15–30	NCI
Gefitinib+cisplatin+RTX	I/II	Locally-advanced	40	AstraZeneca
Gefitinib+RTX±cisplatin	I	Stage III/IV	30	Univ. Colorado
Gefitinib+Paclitaxel+RTX	I	Advanced/recurrent	15–30	NCI
Cisplatin+RTX±Gefitinib concomitant or maintenance	II	Stage III/IV	224	AstraZeneca
				
*Anti-EGFR monoclonal antibodies as monotherapy or in combination with chemotherapy:*				
Cetuximab+albumin−bound paclitaxel (=Abraxane™)	II	Recurrent/metastatic		Univ. California Irvine
Cetuximab+cisplatin or carboplatin and 5-fluorouracil (EXTREME trial)	III	Recurrent/metastatic	440	Merck
				
*Anti-EGFR monoclonal antibodies in combination with radiotherapy:*				
Cisplatin+RTX±Cetuximab	III	Stage III/IV	720	RTOG/NCI
Cetuximab+Pemetrexed+RTX	I	Recurrent	40	Univ. Pittsburg Lilly Bristol-Myers Squibb
Adjuvant Cetuximab+cisplatin vs docetaxel+RTX	II	Stage III/IV	230	RTOG
Cetuximab+cisplatin+RTX	II	Stage III/IV	68	ECOG/NCI
Cetuximab+cisplatin/docetaxel before Cetuximab+cisplatin/RTX	II	Locally-advanced	40	Univ. Pittsburg Bristol-Myers Squibb
Cetuximab+Concomitant-Boost accel. RTX	II	Locally-advanced oropharyngeal	90	Merck

**Table 5:** Rash treatment algorithm (according to Garey *et al*, 2005)

**Grade**	**Macular**	**Pustular**	**Dry**	**Pruritus**	**Ulcerative**
1	Topical steroids	Clindamycin gel	NA	NA	NA
2	Topical steroids	Oral antibiotics	Lotion antihistamine		NA
3	Oral steroids	Oral antibiotics	Lotion antihistamine		Silver sulfadiazine

(consider dermatology consult).
